# Downregulation of SALL1 and its association with poor prognosis and the immune cell landscape in clear cell renal cell carcinoma

**DOI:** 10.3389/fonc.2025.1624651

**Published:** 2025-10-07

**Authors:** Gen-Yi Qu, Shao-Hao Chen, Meng-Xin Liu, Yi-Jie Wang, Zhao-Hui Long, Ning Xu, Xue-Yi Xue

**Affiliations:** ^1^ Department of Urology, Urology Research Institute, The First Affiliated Hospital, Fujian Medical University, Fuzhou, China; ^2^ Department of Urology, National Regional Medical Center, Binhai Campus of the First Affiliated Hospital, Fujian Medical University, Fuzhou, China; ^3^ Department of Urology, Zhuzhou Hospital Affiliated to Xiangya School of Medicine, Central South University, Zhuzhou, China; ^4^ Fujian Key Laboratory of Precision Medicine for Cancer, The First Affiliated Hospital, Fujian Medical University, Fuzhou, China

**Keywords:** SALL1, clear cell renal cell carcinoma (ccRCC), immune microenvironment, drug sensitivity, prognosis, biomarker

## Abstract

**Background:**

Spalt-like transcription factor 1 (SALL1), a member of the Krüppel-associated box-containing zinc finger proteins, has been implicated in tumor suppression, epigenetic regulation, and immune modulation in several malignancies. However, its role in clear cell renal cell carcinoma (ccRCC) remains poorly understood. This study aims to comprehensively assess the clinical significance, immunological relevance, and potential therapeutic value of SALL1 in ccRCC.

**Methods:**

We analyzed transcriptomic and clinical data from public databases to explore the expression, prognostic value, and clinical correlations of SALL1 in ccRCC. The immune cell landscape associated with SALL1 was assessed using various immune algorithms. SALL1 expression in ccRCC tissues and cells was validated by immunohistochemistry (IHC), RT-qPCR, and Western blot analyses. Gene Ontology and KEGG pathway analyses were employed to identify the potential biological functions of SALL1. Functional assays, including MTT, colony formation, and Transwell assays, were performed to evaluate the effects of SALL1 on ccRCC cell proliferation, invasion, and migration. *In vivo* xenograft models using nude mice further supported these findings. We also conducted drug sensitivity analyses using bioinformatic prediction and validated the enhanced sensitivity to Sunitinib in SALL1-overexpressing ccRCC cells through a dose-dependent CCK-8 assay.

**Results:**

SALL1 expression was significantly downregulated in ccRCC tumors compared to normal tissues. Overexpression of SALL1 inhibited cell proliferation, migration, and invasion in A498 and 769-P cell lines. Survival analysis revealed that low SALL1 expression was associated with poor overall survival, progression-free survival, and disease-specific survival. Univariate and multivariate Cox regression analyses confirmed that SALL1 expression is an independent prognostic factor for ccRCC. Functional enrichment analyses indicated that genes associated with SALL1 were enriched in pathways related to ion transport and immune modulation. Furthermore, SALL1 also exhibited positive correlations with the sensitivity to multiple anticancer agents.

**Conclusions:**

SALL1 is significantly downregulated in ccRCC and independently predicts poor clinical outcomes. It exerts tumor-suppressive effects and is involved in shaping an immunologically favorable tumor microenvironment. Functional assays confirmed its ability to enhance drug responsiveness, especially to Sunitinib. These findings suggest that SALL1 may serve as a prognostic biomarker and a potential modulator of immune and therapeutic responses in ccRCC.

## Introduction

Renal cell carcinoma (RCC) represents a significant global health concern, ranking among the top ten most common cancers worldwide. Clear cell renal cell carcinoma (ccRCC), also referred to as kidney renal clear cell carcinoma (KIRC), is the most prevalent subtype, accounting for approximately 70-80% of RCC cases (1–3). Despite advancements in diagnostic methods and treatment options, ccRCC continues to be associated with poor prognosis, primarily due to late-stage diagnosis and the development of resistance to therapeutic interventions (4–6). Current treatment strategies, including nephrectomy, targeted therapies, and immune checkpoint inhibitors (ICIs), have shown varying degrees of success, particularly with PD-1 inhibitors. However, many patients with advanced ccRCC present with metastatic disease or eventually develop resistance to these therapies (7, 8). The tumor microenvironment (TME) has emerged as a critical factor influencing the efficacy of ICIs, with increasing recognition that its composition plays a significant role in the variability of patient responses (9, 10). As a result, there is an urgent need to identify novel biomarkers that can guide prognosis, predict responses to immunotherapy, and facilitate the development of personalized treatment strategies for ccRCC patients.

Zinc finger proteins (ZFPs), crucial transcriptional regulators in the human genome, are predominantly located in the 19q13 region. They play pivotal roles in a range of cellular processes, including gene expression, histodifferentiation, apoptosis, autophagy, and stem cell regulation (11, 12). The SALL1 gene, a member of the SALL gene family, encodes a Krüppel-associated box-containing zinc finger protein (KRAB-ZFP) and has been implicated in the initiation and progression of various human cancers (13, 14). The promoter of SALL1 is frequently methylated in breast cancer and early-stage head and neck cancer, indicating its potential role in tumorigenesis (13, 14). SALL proteins, including SALL1, are essential for organogenesis during embryonic development and play a crucial role in regulating cell proliferation, survival, migration, and stemness, which links them to several cancers and genetic disorders (15, 16). Recent studies have highlighted SALL1’s involvement in colorectal cancer, where it has been identified as a low-risk gene for overall survival. Moreover, its expression is modulated by miR-503–5p, influencing cancer progression (17). Despite its established role in several malignancies, the role of SALL1 in renal cancer, particularly in ccRCC, remains poorly defined. Pan-cancer analyses have shown differential expression of SALL1 in multiple tumor types, including kidney cancer, but a systematic evaluation of its expression profile, prognostic relevance, and biological function in ccRCC is lacking. Additionally, analysis of TCGA-KIRC data suggests that SALL1 is significantly downregulated in ccRCC tissues, implying its potential role as a tumor suppressor. However, no study to date has comprehensively investigated whether SALL1 influences tumor progression, immune regulation, or therapeutic response in the context of ccRCC. Recent advances in transcriptional regulation and epigenetic control have underscored the importance of novel computational tools and mechanisms in uncovering regulatory landscapes in cancer. For example, the TRAPT framework leverages multi-omics epigenomic data to accurately predict transcriptional regulators and could serve as a valuable reference for understanding upstream regulators of SALL1 and its associated pathways in ccRCC (18). Moreover, studies have shown that DHX16-mediated ribosome assembly plays an essential role in stem cell maintenance and may intersect with SALL1-mediated regulation (19). In addition, immune-related regulation involving IL-1β/NF-κB/miR-506/JAG1 signaling (20) and JAK/STAT3 signaling modulation (21) offer insight into the broader immunologic networks potentially connected to SALL1 function in the tumor microenvironment. These insights further support a need to explore whether SALL1 contributes to immune landscape modulation in ccRCC.

In this study, we systematically investigate the expression levels of SALL1 in ccRCC and its association with clinical outcomes. Additionally, we explore the regulatory mechanisms of SALL1, with particular focus on its involvement in immune-related pathways and its impact on the tumor microenvironment. By analyzing the relationship between SALL1 expression and immune cell infiltration, we aim to uncover its potential as both a prognostic marker and a therapeutic target in ccRCC. Understanding these mechanisms may offer new insights into ccRCC progression and open avenues for the development of more effective treatment strategies.

## Methods

### Data collection

Clinical and mRNA expression data for clear cell renal cell carcinoma (ccRCC) were obtained from The Cancer Genome Atlas Kidney Renal Clear Cell Carcinoma (TCGA-KIRC) cohort via the Genomic Data Commons (https://portal.gdc.cancer.gov/). Only samples classified under the KIRC subtype were included in the analysis. Additional datasets, including GSE53757 and GSE66272, were retrieved from the Gene Expression Omnibus (GEO) using the “GEO2R” tool, providing mRNA expression data for 99 specimens. Clinical data for these patients were integrated into the study and can be accessed via the TCGA portal (https://portal.gdc.cancer.gov/). Furthermore, tissue samples were collected from 30 ccRCC patients who underwent surgical resection at The First Affiliated Hospital of Fujian Medical University between January 2024 and June 2024. None of these patients received systemic or local treatments, including chemotherapy or radiotherapy, prior to surgery. Matched tumor and adjacent normal tissues were immediately stored at −80 °C for long-term preservation until RNA and protein extraction. Ethical approval for this study was granted by the Ethics Committee of The First Affiliated Hospital of Fujian Medical University, and all patients provided written informed consent for participation.

### Differential expression analysis of SALL1 in ccRCC

The Tumor Immune Estimation Resource (TIMER) database (TIMER2.0, http://timer.comp-genomics.org/) was used to examine the mRNA expression levels of SALL1 across multiple cancer types, including ccRCC. The Diff Exp module in TIMER2.0 was applied to compare SALL1 expression between tumor and normal tissues, with statistical significance assessed using the Wilcoxon test. Additionally, independent ccRCC cohorts were downloaded from the GEO database and analyzed using GEO2R to validate the differential expression of SALL1 between normal and tumor tissues.

### Clinicopathological and prognostic analysis

To assess the clinical significance of SALL1 in ccRCC, its association with various clinicopathological parameters, including age, sex, tumor grade, and TNM staging, was analyzed using data from TCGA. Kaplan-Meier survival curves were generated to evaluate the impact of SALL1 expression on overall survival (OS), progression-free survival (PFS), and disease-specific survival (DSS) in ccRCC patients. Additionally, univariate and multivariate Cox regression analyses were conducted to determine whether SALL1 expression serves as an independent prognostic factor, adjusting for age, tumor grade, tumor stage, and metastatic status. The analyses were performed using the “survival” and “survminer” packages in R, and hazard ratios (HRs) with 95% confidence intervals (CIs) were calculated.

### Gene co-expression and functional enrichment analysis

To explore the molecular interactions of SALL1 in ccRCC, co-expressed genes were identified using the “limma” package (version 3.52.4), a widely used tool for differential expression analysis in gene expression studies. We applied a statistical threshold of adjusted P-value < 0.05 and |log2(Fold Change)| > 1 to define significantly co-expressed genes, consistent with the approach used in published differential expression studies (22). Functional enrichment analysis was performed using the “clusterProfiler” package (version 4.4.4), which enables statistical analysis and visualization of functional profiles for genes and gene clusters (23). Gene Ontology (GO) analysis was used to categorize genes into biological processes, molecular functions, and cellular components, while Kyoto Encyclopedia of Genes and Genomes (KEGG) pathway analysis was performed to identify enriched signaling pathways. Enrichment results were considered statistically significant at adjusted P < 0.05. Visualization of enrichment results was performed using functions from the “enrichplot” and “ggplot2” packages.

### Immune cell infiltration analysis

To examine the association between SALL1 expression and immune cell infiltration in ccRCC, immune cell composition data were extracted from tumor samples and statistically analyzed. The correlation between SALL1 levels and immune invasion scores was determined using the “psych” package in R. Additionally, the CIBERSORT algorithm, integrated with the “limma” and “tidyverse” packages, was used to profile the tumor immune microenvironment by quantifying the infiltration levels of distinct immune cell subsets. We further applied multiple immune deconvolution algorithms, including TIMER2.0 and MCP-counter. TIMER2.0 was used to estimate the infiltration levels of six major immune cell types. MCP-counter was employed via the “MCPcounter” R package to quantify the abundance of immune and stromal populations in tumor tissues. All calculations were performed using normalized RNA-seq data from TCGA-KIRC cohort. Spearman’s or Pearson’s correlation coefficients were computed to assess the associations between SALL1 expression and immune cell infiltration scores.

### Drug response analysis

Drug sensitivity patterns were assessed using the “pRRophetic” package, with results graphically represented through “ggplot2”. This analysis explored the association between SALL1 expression and responsiveness to various anticancer agents, including tyrosine kinase inhibitors and chemotherapy drugs.

### Cell culture

Human ccRCC cell lines (Caki-2, A498, 769-P, and OS-RC-2) and normal renal epithelial cells (HK-2) were obtained from the American Type Culture Collection (ATCC). Cells were cultured in Dulbecco’s Modified Eagle’s Medium (DMEM) (Gibco, Thermo Fisher Scientific) supplemented with 10% fetal bovine serum (FBS) and 1% penicillin-streptomycin (Gibco). Cells were cultured in a humidified incubator at 37 °C with 5% CO_2_ to ensure stable conditions for optimal cell growth and viability.

### Plasmid construction and transfection

The pcDNA3.1 vectors containing the full-length SALL1 gene were sourced from RioBio (Guangzhou, China). ccRCC cells were plated into 6-well plates at a density of 2 × 10^5^ cells per well and allowed to adhere for 24 hours to reach approximately 60–70% confluence at the time of transfection. Each well was transfected with 2 µg of plasmid DNA using Lipofectamine 3000 (Invitrogen, Carlsbad, CA, USA) at a DNA:reagent ratio of 1:2, following the manufacturer’s protocol. After 48 hours, stable transfectants were selected by treatment with 2 μg/mL puromycin (Sigma, St. Louis, MO, USA) for one week to ensure the establishment of stable cell lines. The overexpression efficiency was validated at both mRNA and protein levels using qRT-PCR and Western blotting, respectively.

### RNA extraction and quantitative real-time PCR

Total RNA from cells and tissues was extracted using TRIzol^®^ Reagent (Invitrogen, Thermo Fisher Scientific, USA) according to the manufacturer’s instructions. The purity and concentration of RNA were determined using a NanoDrop 2000 spectrophotometer (Thermo Fisher Scientific). RNA integrity was assessed by 1% agarose gel electrophoresis and visualization of intact 28S and 18S rRNA bands. Samples with A260/A280 ratios between 1.8 and 2.0 were considered of acceptable quality. Complementary DNA (cDNA) was synthesized using the Transcriptor First Strand cDNA Synthesis Kit (Roche Diagnostics, Basel, Switzerland). For each reaction, 1 μg of total RNA was used in a 20 μL reaction system, following the manufacturer’s protocol. The cDNA synthesis reaction was performed at 55 °C for 30 minutes followed by 85 °C for 5 minutes to inactivate the reverse transcriptase. Synthesized cDNA was stored at −20 °C until further use. Quantitative real-time PCR (qRT-PCR) was performed on an ABI 7300 real-time PCR system (Applied Biosystems, Thermo Fisher Scientific) using FastStart Universal SYBR^®^ Green Mix (Roche Diagnostics). The specific primer sequences for SALL1 were: SALL1 F: 5’-TGATGTAGCCAGCATGT-3’, R: 5’-AAAGAATTCAGCGCAGCAC-3’. β-actin was used as an internal control, and relative gene expression levels were analyzed using the 2^−ΔΔCt method.

### Western blot

Total protein was extracted using RIPA lysis buffer (Beyotime, China) supplemented with protease inhibitors. Protein concentration was quantified using a BCA protein assay kit (Beyotime). Equal amounts of protein were loaded onto a 10% SDS-PAGE gel, separated by electrophoresis, and transferred onto PVDF membranes. The membranes were blocked with 5% non-fat milk and incubated overnight at 4 °C with primary antibodies, including SALL1 (ab31526, Abcam, Cambridge, MA, USA). After washing, the membranes were incubated for 1 hour at room temperature with HRP-conjugated secondary antibodies (#7074, #7076, 1:1000, Cell Signaling Technology, Danvers, MA, USA). Protein bands were detected using an ECL Plus detection kit (Pierce, Rockford, IL, USA), and densitometric analysis was performed using ImageJ software (National Institutes of Health, Bethesda, MD, USA). β-actin served as a loading control.

### Immunohistochemistry

Immunohistochemical staining was performed following established protocols. Tissue sections were incubated overnight at 4 °C with a SALL1 primary antibody (1:1500, ab41974, Abcam, Cambridge, MA, USA). Staining intensity was classified into four levels (0–3), representing no, weak, moderate, and strong staining, respectively. The percentage of positively stained cancer cells was graded on a scale of 1–4, corresponding to 0–25%, 25–50%, 50–75%, and ≥75%. The final IHC score was determined by multiplying the staining intensity and positive cell percentage scores.

### Cell viability assay

To assess cell viability following drug treatment, a CCK-8 assay was performed. Approximately 5,000 cells per well were seeded into 96-well plates and incubated overnight. Cells were then treated with different concentrations of Sunitinib for 48 hours. After treatment, 10% CCK-8 reagent (v/v) was added to each well and incubated for 2 hours at 37 °C. Absorbance was measured at 450 nm using a microplate reader. Cell viability was calculated based on absorbance values relative to untreated controls.

### MTT assay

Transfected ccRCC cells were seeded at a density of 1 × 10³ cells per well in 96-well plates and incubated for 0 h, 24 h, 48 h, and 72 h. At each time point, 10 μL of MTT solution (0.1 mg/mL, Sigma) was added to each well and incubated at 37 °C for 4 hours. The culture medium was then carefully removed, and 150 μL of DMSO was added to each well to dissolve the formazan crystals. The absorbance was measured at 570 nm using a microplate reader (Bio-Rad Laboratories Inc., Hercules, CA, USA) to assess cell proliferation rates. Each group included three biological replicates (n = 3), and all experiments were repeated independently at least three times. Data were analyzed using two-way ANOVA followed by Bonferroni correction for multiple comparisons.

### EdU assay

Transfected ccRCC cells were seeded into 24-well plates at a density of 1 × 10^5^ cells per well and incubated overnight. The cells were then treated with 50 μM 5-Ethynyl-2′-deoxyuridine (EdU, Beyotime, Shanghai, China) for 2 hours at 37 °C to assess DNA synthesis. After incubation, the cells were fixed with 4% paraformaldehyde for 10 minutes and stained with Apollo fluorochrome. Nuclear staining was performed using DAPI, and the proportion of EdU-positive cells was analyzed under a confocal microscope (Olympus, Tokyo, Japan).

### Colony formation assay

ccRCC cells were plated at a density of 1000 cells per well in 6-well plates and cultured at 37 °C with 5% CO_2_ in a humidified incubator for 14 days under conditions favorable for colony formation, with medium refreshed every 3 days. After incubation, the plates were washed with PBS, and the colonies were fixed with 4% formaldehyde for 30 minutes. The colonies were then stained with 0.2% crystal violet solution at room temperature for 30 minutes. The colonies were photographed and counted under a microscope (Olympus). Colony number was quantified from at least three randomly chosen fields per well, across three independent biological replicates. Data were presented as mean ± SD, and statistical significance was assessed using unpaired Student’s t-test.

### Transwell assay

A 24-well Transwell system with 8-μm pore size membranes (BD Biosciences, San Jose, CA, USA) was used to assess migration and invasion abilities of transfected ccRCC cells. Prior to the assay, cells were serum-starved for 8 hours in serum-free medium. Subsequently, 4 × 10^4^ cells suspended in 500 μL serum-free medium were added to the upper chamber, which was either uncoated (for migration) or pre-coated with Matrigel (for invasion). The lower chamber was filled with 700 μL of culture medium containing 20% FBS as a chemoattractant. After 24 hours, non-migrated cells were discarded, and the cells on the lower surface of the membrane were fixed with 4% paraformaldehyde and stained with 0.1% crystal violet. Migrated or invaded cells were counted in five randomly selected fields under a microscope (Olympus). For each group, at least three independent biological replicates were performed. Migrated and invaded cells were counted in five randomly selected fields per membrane using ImageJ software. Statistical differences between groups were analyzed using unpaired Student’s t-test.

### Animal experiments

Six-week-old BALB/c nude mice weighing 20 ± 2 g were purchased from Beijing Vital River Laboratory Animal Technology Co., Ltd. (Beijing, China). The mice were randomly assigned into two groups: vector control and oe-SALL1 (n = 4 per group). The 769-P cells stably transfected with either a vector or oe-SALL1 (5 × 10^6^ cells per mouse) were subcutaneously injected into the axilla. Tumor size was measured weekly, and after 4 weeks, the mice were euthanized, and the tumors were excised for weight measurement. The excised tumor tissues were fixed in 4% paraformaldehyde for 24 hours and processed for hematoxylin and eosin (HE) staining. All animal experiments were reviewed and approved by the Animal Care and Use Committee of The Second Xiangya Hospital, Central South University (Project License No. 2023104). The study was conducted in compliance with institutional guidelines and the National Institutes of Health Guide for the Care and Use of Laboratory Animals. Mice were housed under standard conditions (temperature 22 ± 2 °C, relative humidity 50% ± 10%, 12-hour light/dark cycle) with free access to food and water. The sample size (n = 4 per group) was selected based on previous pilot data and literature precedent to observe meaningful tumor growth differences within a limited animal cohort, while minimizing animal usage in accordance with the 3Rs (Replacement, Reduction, Refinement) principles.

### Statistical analysis

Statistical analyses were conducted using R software (v4.2.2). For continuous variables, group comparisons were made using the Student’s t-test or one-way ANOVA, as appropriate. Kaplan-Meier survival curves were analyzed with the log-rank test, and Pearson’s chi-square test was used to assess correlations between SALL1 expression and clinicopathological parameters. Univariate and multivariate Cox regression analyses were performed to identify independent prognostic factors. All experiments were repeated at least three times independently to ensure reproducibility. Statistical calculations were performed using GraphPad Prism 9.0 (GraphPad, San Diego, CA, USA), and a P-value < 0.05 was considered statistically significant.

## Results

### Expression of SALL1 in pan-cancer

**Figure 1:**
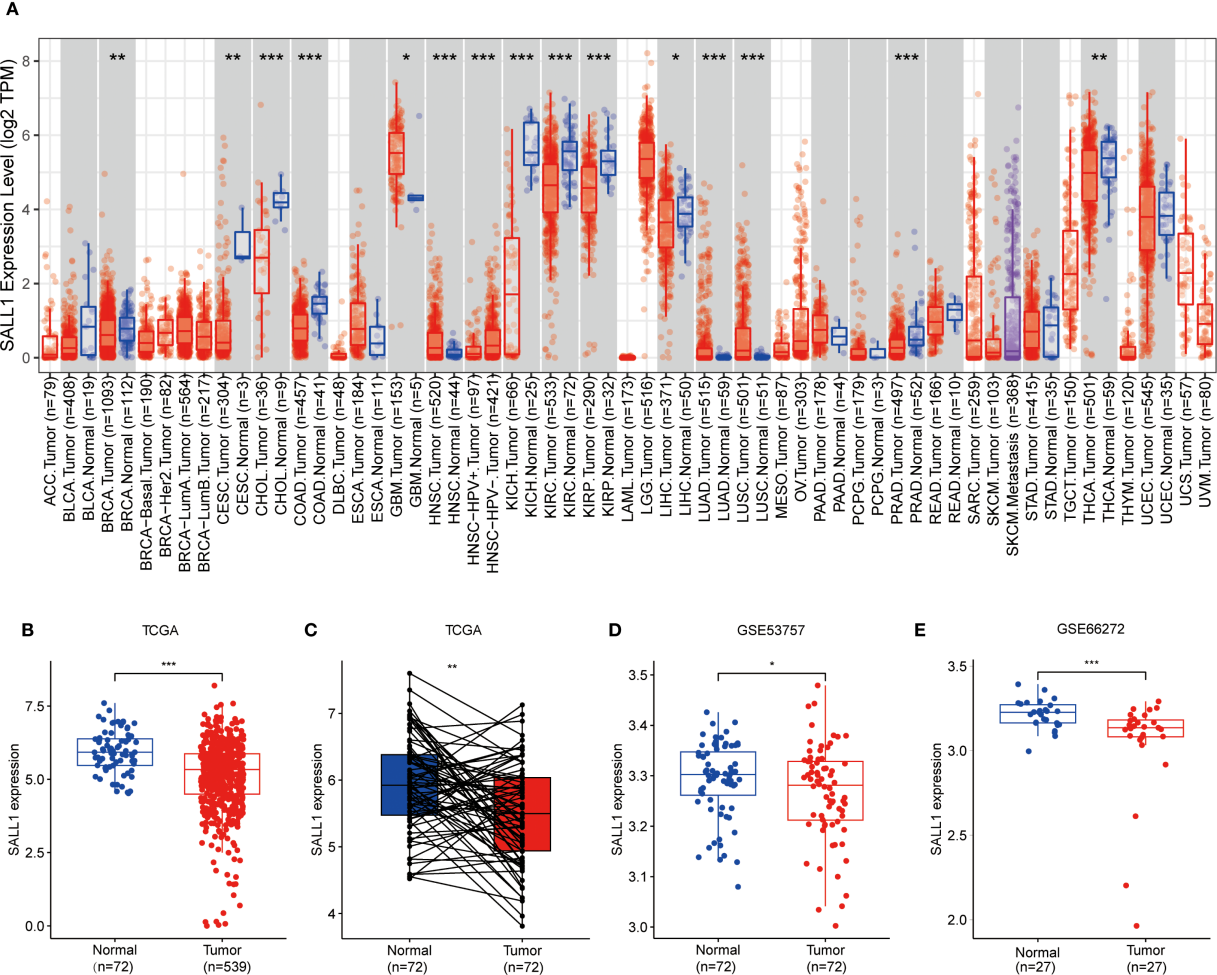
Downregulation of SALL1 in ccRCC. **(A)** Boxplot illustrating SALL1 expression across various cancer types from the TCGA dataset. Tumor samples are depicted in red, while normal tissues are represented in blue (*P < 0.05, **P < 0.01, ***P < 0.001. ACC, Adrenocortical carcinoma; BLCA, Bladder urothelial carcinoma; BRCA, Breast invasive carcinoma; CESC, Cervical squamous cell carcinoma and endocervical adenocarcinoma; CHOL, Cholangiocarcinoma; COAD, Colon adenocarcinoma; DLBC, Lymphoid Neoplasm Diffuse Large B-cell Lymphoma; ESCA, Esophageal carcinoma; GBM, Glioblastoma multiforme; HNSC, Head and neck squamous cell carcinoma; KICH, Kidney chromophobe; KIRC, Kidney renal clear cell carcinoma; KIRP, Kidney renal papillary cell carcinoma; LAML, Acute myeloid leukemia; LGG, Brain lower grade glioma; LIHC, Liver hepatocellular carcinoma; LUAD, Lung adenocarcinoma; LUSC, Lung squamous cell carcinoma; MESO; Mesothelioma; OV, Ovarian serous cystadenocarcinoma; PAAD, Pancreatic adenocarcinoma; PCPG, Pheochromocytoma and paraganglioma; PRAD, Prostate adenocarcinoma; READ, Rectum adenocarcinoma; SARC; Sarcoma; SKCM, Skin cutaneous melanoma; STAD, Stomach adenocarcinoma; TGCT, Testicular germ cell tumor; THCA, Thyroid carcinoma; THYM; Thymoma; UCEC, Uterine corpus endometrial carcinoma; UCS, Uterine carcinosarcoma; UVM, Uveal melanoma). **(B, C)** SALL1 expression in ccRCC from TCGA. **(B)** Comparison of normal (n = 72) and tumor (n = 539) samples. **(C)** Paired analysis of matched tumor and normal samples (n = 72). **(D, E)** Validation of SALL1 downregulation in ccRCC using the GSE53757 (n = 72 per group) and GSE66272 (n = 27 per group) datasets.

To explore how SALL1 is expressed across various cancer types, we used the TIMER2.0 tool and analyzed data from the TCGA database. The results showed that SALL1 was notably downregulated in several cancers, including CHOL (cholangiocarcinoma), COAD (colon adenocarcinoma), KICH (kidney chromophobe), KIRP (kidney renal papillary cell carcinoma), KIRC (kidney renal clear cell carcinoma), and PRAD (prostate adenocarcinoma) (P < 0.001). Additionally, cancers like BRCA (breast invasive carcinoma), CESC (cervical squamous cell carcinoma), and THCA (thyroid carcinoma) showed significant downregulation (P < 0.01), with LIHC (liver hepatocellular carcinoma) showing a lesser degree of downregulation (P < 0.05, **Figure 1A**).

### Downregulation of SALL1 in ccRCC tissues and cell lines

In the TCGA cohort, SALL1 expression was significantly lower in ccRCC tumor samples (n = 539) compared to normal tissues (n = 72) (**Figure 1B**). A paired analysis of tumor and normal tissue samples (**Figure 1C**) confirmed this decrease in SALL1 expression in ccRCC. Further validation was done using datasets GSE53757 and GSE66272 (**Figures 1D, E**), where tumor tissues showed significantly lower SALL1 levels than normal kidney tissues. This supports the idea that SALL1 downregulation is linked to ccRCC progression, possibly suggesting its role as a tumor suppressor.

**Figure 2:**
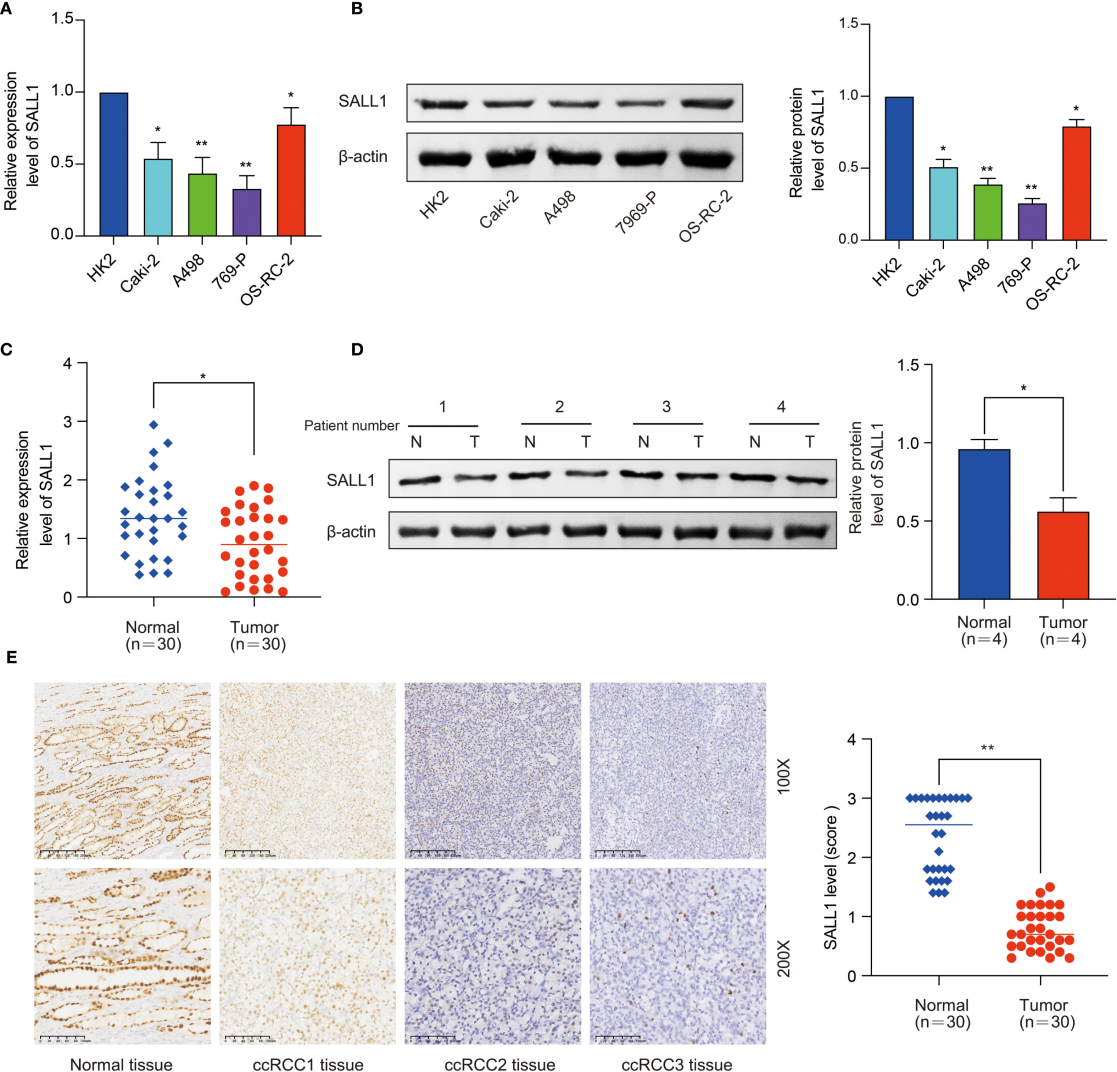
Downregulation of SALL1 in ccRCC cell lines and tissues. **(A)** Relative mRNA expression levels of SALL1 in human renal cell lines (HK2, Caki-2, A498, 769-P, OS-RC-2), normalized to the HK2 cell line (*P < 0.05, **P < 0.01). **(B)** Western blot analysis of SALL1 protein expression in the same cell lines (*P < 0.05, **P < 0.01). **(C)** Relative mRNA expression of SALL1 in paired tumor (T) and normal (N) tissues from 30 ccRCC patients (*P < 0.05). **(D)** Western blot analysis of SALL1 expression in paired tumor and normal tissues from ccRCC patients (*P < 0.05). **(E)** Immunohistochemical staining of SALL1 in normal kidney tissue and ccRCC tissues(*P < 0.01).

Further studies in renal cell lines showed that SALL1 mRNA levels were significantly lower in Caki-2, A498, 769-P, and OS-RC-2 cell lines compared to the normal HK2 cell line (*P < 0.05, **Figure 2A**). All qPCR reactions were normalized to β-actin, and three biological replicates were performed per group. This finding was confirmed by Western blot analysis (**Figure 2B**), which showed reduced SALL1 protein levels in these cancer cell lines compared to HK2 (*P < 0.05), β-actin served as a loading control. We also analyzed SALL1 expression in paired tumor and normal tissues from 30 ccRCC patients, and found that SALL1 mRNA was significantly lower in tumor tissues (*P < 0.05, **Figure 2C**). In four randomly selected paired samples, Western blotting further confirmed reduced SALL1 protein in tumors (**Figure 2D**), with statistical analysis showing a significant reduction (p < 0.05). Again, β-actin served as the internal control for normalization. Immunohistochemistry (IHC) was performed on formalin-fixed paraffin-embedded tissues from 30 patients. Representative images (**Figure 2E**, 100× and 200×) revealed markedly lower SALL1 staining in tumor tissues compared with normal counterparts. Semi-quantitative scoring of staining intensity revealed significantly decreased SALL1 expression in tumor tissues (*P < 0.001), with a median IHC score of 0.79 compared to 2.34 in normal tissues. These results emphasize that SALL1 downregulation is a key feature in ccRCC and may contribute to its development.

### Association between SALL1 expression and clinical characteristics in ccRCC

**Figure 3:**
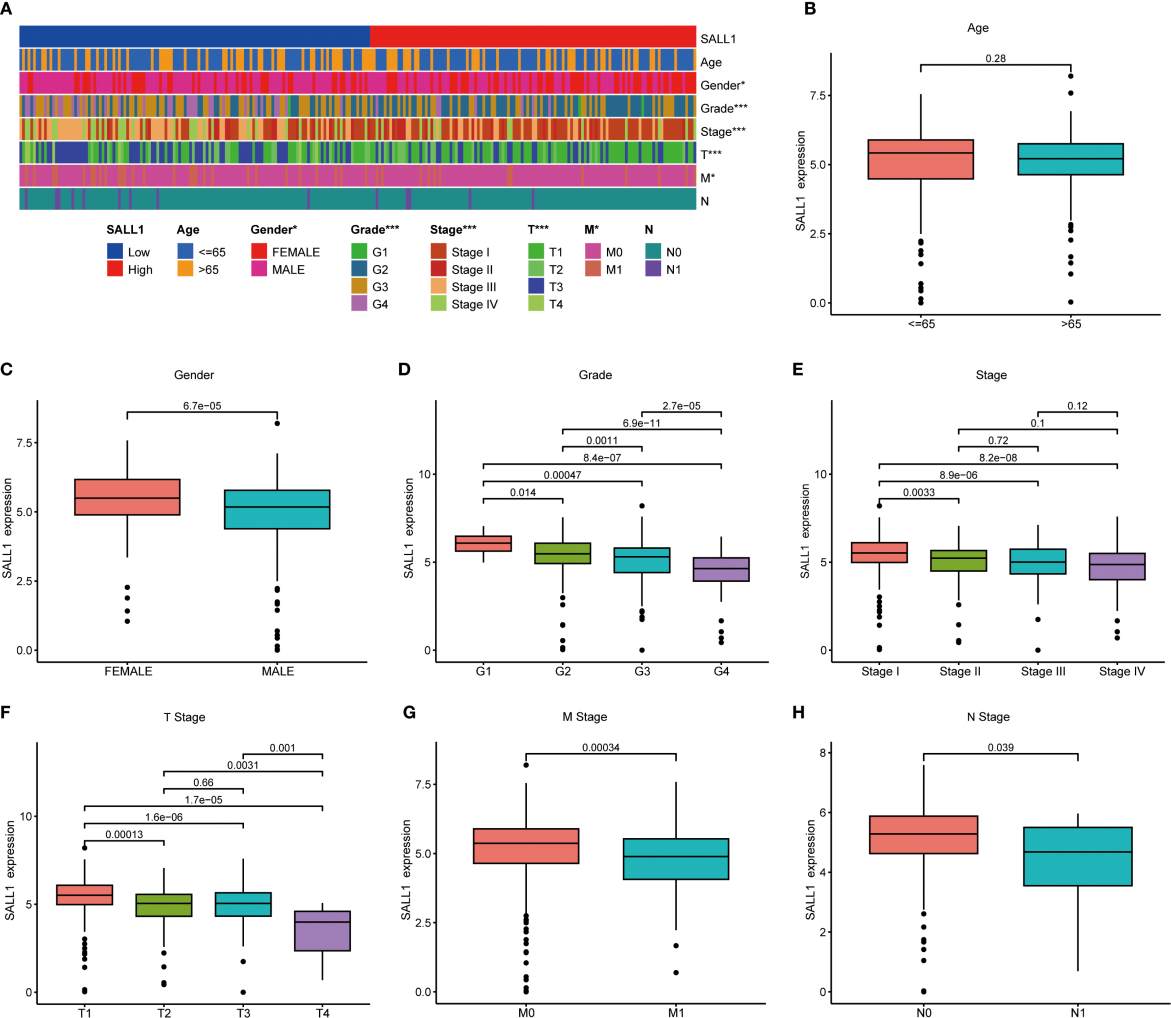
Correlation between SALL1 expression and clinical parameters in ccRCC. **(A)** Heatmap showing the correlation of SALL1 expression with age, gender, tumor grade, and TNM stage (*P < 0.05, ***P < 0.001). **(B-H)** Boxplots illustrating the relationship between SALL1 expression and various clinical parameters: **(B)** Age, **(C)** Gender, **(D)** Tumor grade, **(E)** Tumor stage, **(F)** T stage, **(G)** M stage, **(H)** N stage.

To better understand the relationship between SALL1 expression and clinical characteristics in ccRCC, we analyzed data from TCGA. A heatmap (**Figure 3A**) showed the distribution of SALL1 expression across various clinical parameters, including age, sex, tumor grade, and TNM stage. There was no significant difference in SALL1 expression between patients aged ≤65 and >65 years (P = 0.28, **Figure 3B**). However, male patients had significantly lower SALL1 expression compared to females (P = 6.7e-05, **Figure 3C**). Additionally, lower SALL1 expression was associated with higher tumor grades (G2-G4) (P < 0.05, **Figure 3D**) and more advanced tumor stages (Stage III-IV) (P < 0.05, **Figure 3E**). Similarly, SALL1 expression was notably reduced in tumors with higher T stages (T2-T4, P < 0.05, **Figure 3F**), as well as in patients with lymph node metastasis (N1, P = 0.039, **Figure 3H**) and distant metastasis (M1, P = 0.00034, **Figure 3G**). These findings suggest that SALL1 downregulation is strongly linked to tumor progression and metastasis in ccRCC.

### Prognostic value of SALL1 in ccRCC

**Figure 4:**
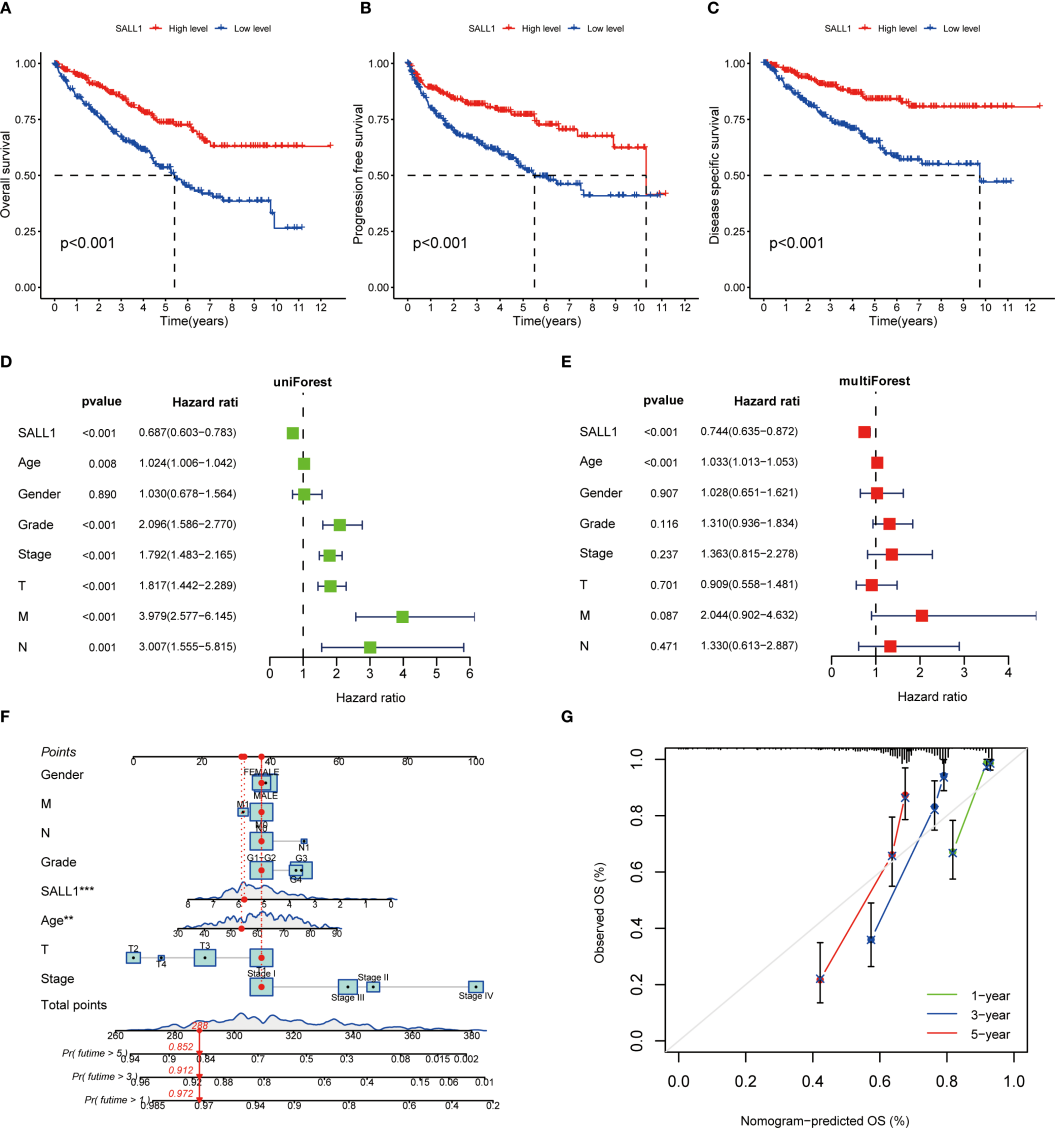
Prognostic value of SALL1 in ccRCC. **(A-C)** Kaplan-Meier survival analysis demonstrating the effect of SALL1 expression on survival outcomes in ccRCC patients: **(A)** OS, **(B)** PFS, **(C)** DSS (P < 0.001). **(D, E)** Forest plots of univariate **(D)** and multivariate **(E)** Cox regression analyses identifying SALL1 expression as an independent prognostic factor in ccRCC. **(F)** Nomogram predicting 1-year, 3-year, and 5-year OS based on SALL1 expression and clinical factors. **(G)** Calibration curve assessing the predictive accuracy of the nomogram for 1-year, 3-year, and 5-year OS in ccRCC patients.

The Kaplan-Meier survival analysis showed that low levels of SALL1 expression were strongly linked to worse outcomes in ccRCC patients. Those with lower SALL1 expression had significantly poorer overall survival (OS), progression-free survival (PFS), and disease-specific survival (DSS) compared to those with higher SALL1 levels (P < 0.001, **Figures 4A–C**). This suggests that the downregulation of SALL1 could be a contributing factor to disease progression and unfavorable clinical outcomes. Both univariate and multivariate Cox regression analyses confirmed that SALL1 expression remains an independent prognostic factor in ccRCC, even when adjusted for clinical variables like age, tumor grade, stage, and metastasis (**Figures 4D, E**). A nomogram predicting OS at 1, 3, and 5 years was constructed using SALL1 expression and clinical factors (**Figure 4F**). The calibration curve indicated a strong alignment between predicted and observed survival probabilities, emphasizing the reliability of the nomogram in predicting ccRCC survival (**Figure 4G**). These results highlight SALL1’s potential as a prognostic biomarker for ccRCC.

### Functional enrichment and immune microenvironment analysis of SALL1 in ccRCC

**Figure 5:**
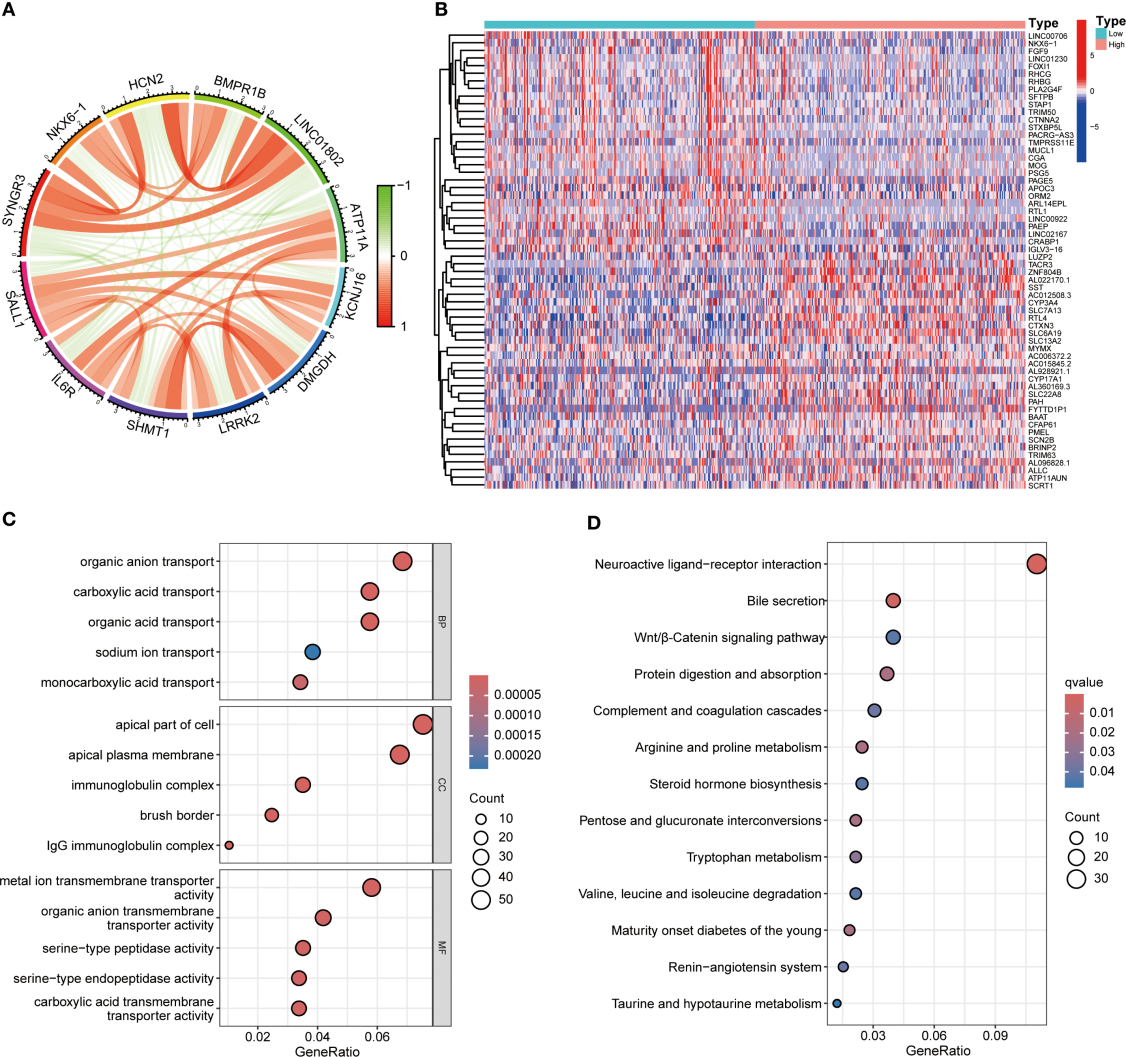
Functional enrichment analysis of SALL1-associated genes in ccRCC. **(A)** Circos plot showing interactions between SALL1 and its associated genes. **(B)** Heatmap of differentially expressed genes (DEGs) in ccRCC patients stratified by SALL1 expression levels (low vs. high). **(C)** GO enrichment analysis highlighting the top enriched biological processes in SALL1-associated genes. **(D)** KEGG pathway enrichment analysis of SALL1-associated genes.

**Figure 6:**
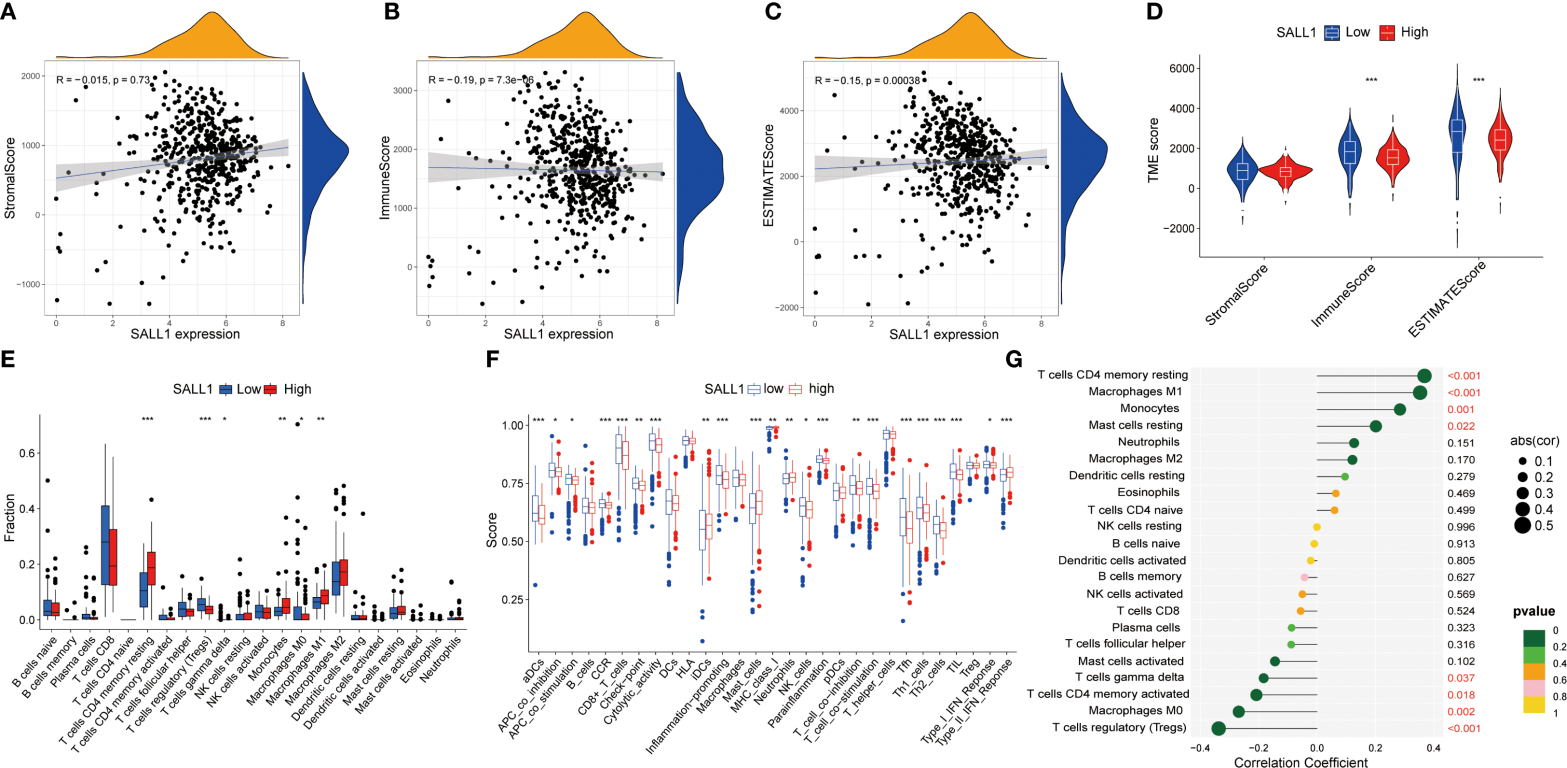
Association between SALL1 expression and tumor immune microenvironment in ccRCC. **(A-C)** Correlation analysis between SALL1 expression and various tumor microenvironment TME scores: **(A)** StromalScore, **(B)** ImmuneScore, and **(C)** ESTIMATEScore. **(D)** Violin plot showing SALL1 expression and its correlation with TME scores. **(E)** Boxplot comparing immune cell fraction in high vs. low SALL1 expression groups (***P < 0.001). **(F)** Immune infiltration analysis comparing immune cell scores in high vs. low SALL1 expression groups. **(G)** Correlation of SALL1 expression with immune-related factors (*P < 0.05, **P < 0.01, ***P < 0.001).

Functional enrichment analysis of genes related to SALL1 in ccRCC showed significant involvement in various biological processes and pathways. **Figure 5A** illustrates the Circos plot, highlighting both positive and negative correlations between SALL1 and its associated genes. The heatmap in **Figure 5B** shows differential gene expression between high and low SALL1 groups, with many genes related to ion transport and immune functions showing significant expression changes. Among the ion transport-related genes, we identified SLC13A2, SLC7A13, SLC6A19, SCN2B, and SLC22A8 as significantly differentially expressed between high and low SALL1 groups. For immune-related genes, representative examples include CGA, ORM2, PAEP, and PAGE5. **Figures 5C, D** present the GO and KEGG enrichment in detail: in GO–BP, genes were enriched in organic anion/carboxylic acid/monocarboxylic acid transport and sodium/metal-ion transport; in GO–CC, enrichment involved the apical part of cell, apical plasma membrane, brush border, and immunoglobulin (IgG) complex; in GO–MF, metal-ion transmembrane transporter activity, organic anion transmembrane transporter activity, serine-type peptidase/endopeptidase activity, and carboxylic acid transmembrane transporter activity were highlighted. KEGG analysis indicated significant pathways including neuroactive ligand–receptor interaction, bile secretion, Wnt/β-Catenin signaling, protein digestion and absorption, complement and coagulation cascades, arginine and proline metabolism, steroid hormone biosynthesis, pentose and glucuronate interconversions, tryptophan metabolism, branched-chain amino-acid degradation, maturity-onset diabetes of the young, renin–angiotensin system, and taurine/hypotaurine metabolism, linking SALL1 to tubular transport/polarity, immune–inflammatory programs, and metabolic rewiring relevant to ccRCC progression and therapy response. In immune microenvironment analysis (**Figure 6**), SALL1 was found to significantly influence immune cell infiltration in ccRCC. **Figures 6A–C** revealed a negative correlation between SALL1 expression and both ImmuneScore and ESTIMATEScore, suggesting a more immune-suppressive environment in tumors with low SALL1 expression. **Figures 6E, F** show that higher SALL1 expression was associated with greater infiltration of immune cells like M1 macrophages and resting CD4 memory T cells. Additionally, SALL1 was linked to various immune checkpoints and cell types (**Figure 6G**), implying a role in modulating the immune response and possibly contributing to immune evasion in ccRCC. These findings underscore the important impact of SALL1 on both tumor metabolism and the immune landscape in ccRCC.

### Association of SALL1 expression with immune checkpoints and drug sensitivity in ccRCC

**Figure 7:**
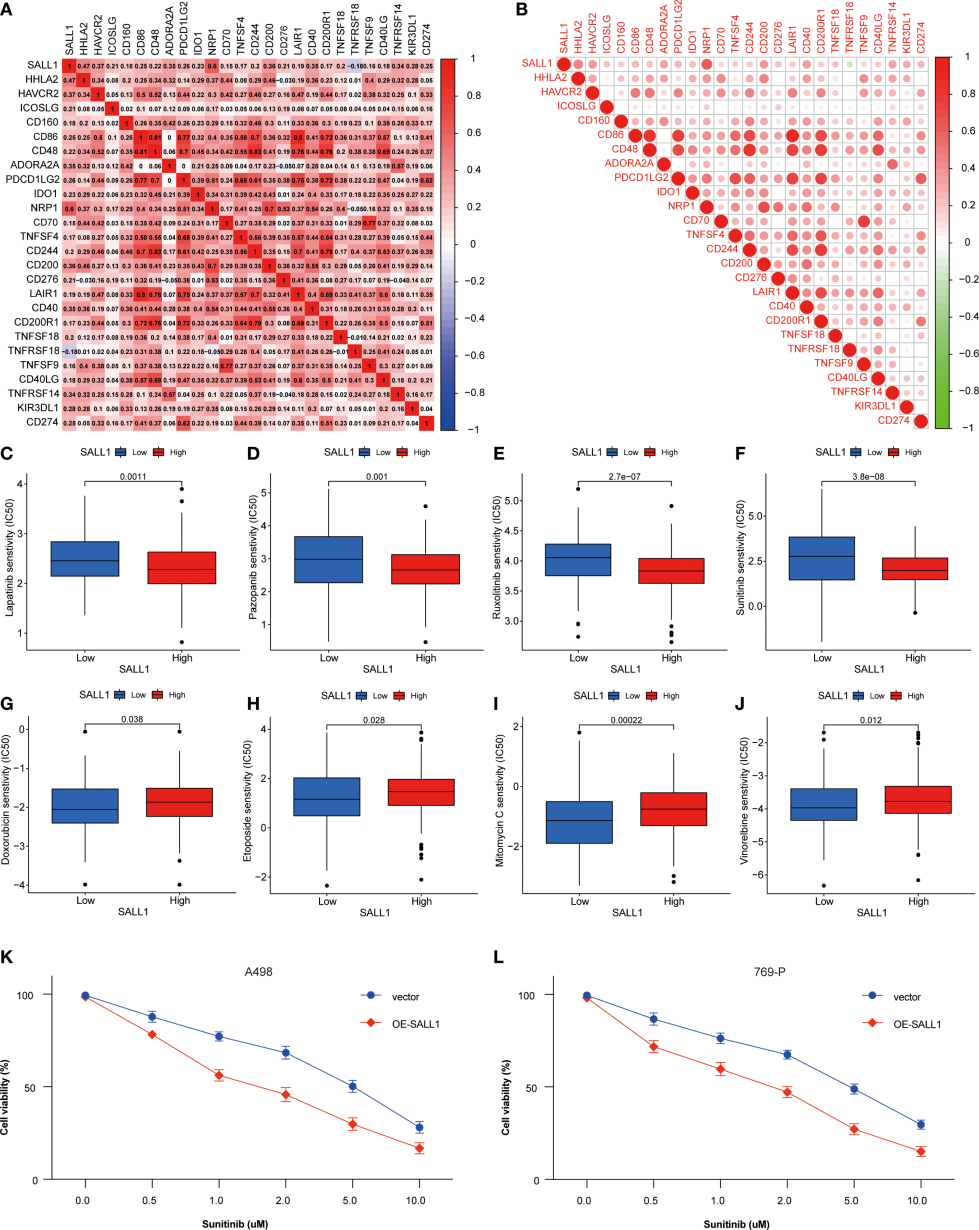
Association between SALL1 expression, immune checkpoints, and drug sensitivity in ccRCC. **(A-B)** Correlation matrix illustrating the relationship between SALL1 expression and immune checkpoint genes. **(C-J)** Boxplots showing the association of SALL1 expression with sensitivity to different anticancer drugs: **(C)** Lapatinib, **(D)** Pazopanib, **(E)** Rucaparib, **(F)** Sunitinib, **(G)** Doxorubicin, **(H)** Epirubicin, **(I)** Mitomycin C, **(J)** Vincristine. **(K, L)** Functional validation of drug sensitivity to Sunitinib was performed using ccRCC cell lines (A498 and 769-P) overexpressing SALL1 (OE-SALL1).

SALL1 expression showed a positive correlation with several immune checkpoint genes, including PD-1 (PDCD1), PD-L1 (CD274), and CTLA4, indicating a potential role in immune modulation in ccRCC (**Figures 7A, B**). Moreover, SALL1 expression was associated with immune cell markers like CD80 and CD40, which further suggests its involvement in immune responses. High SALL1 expression also correlated with increased sensitivity to several anticancer drugs, as shown in **Figures 7C–J**. Boxplots demonstrated that SALL1 overexpression increased the sensitivity of ccRCC cells to Lapatinib, Pazopanib, Rucaparib, Sunitinib, Doxorubicin, Epirubicin, Mitomycin C, and Vincristine. To functionally validate this correlation, we performed dose-response assays using A498 and 769-P ccRCC cell lines with SALL1 overexpression (OE-SALL1). As shown in **Figures 7K, L**, OE-SALL1 cells exhibited significantly reduced cell viability across increasing concentrations of Sunitinib compared to control vector-transfected cells, demonstrating enhanced drug sensitivity. These results suggest that SALL1 not only correlates with immune checkpoint gene expression but also causally contributes to increased chemosensitivity, particularly to Sunitinib. Therefore, SALL1 may serve as both a predictive biomarker and a potential therapeutic modulator in ccRCC.

### Functional role of SALL1 in proliferation, migration, and invasion in ccRCC cells

**Figure 8:**
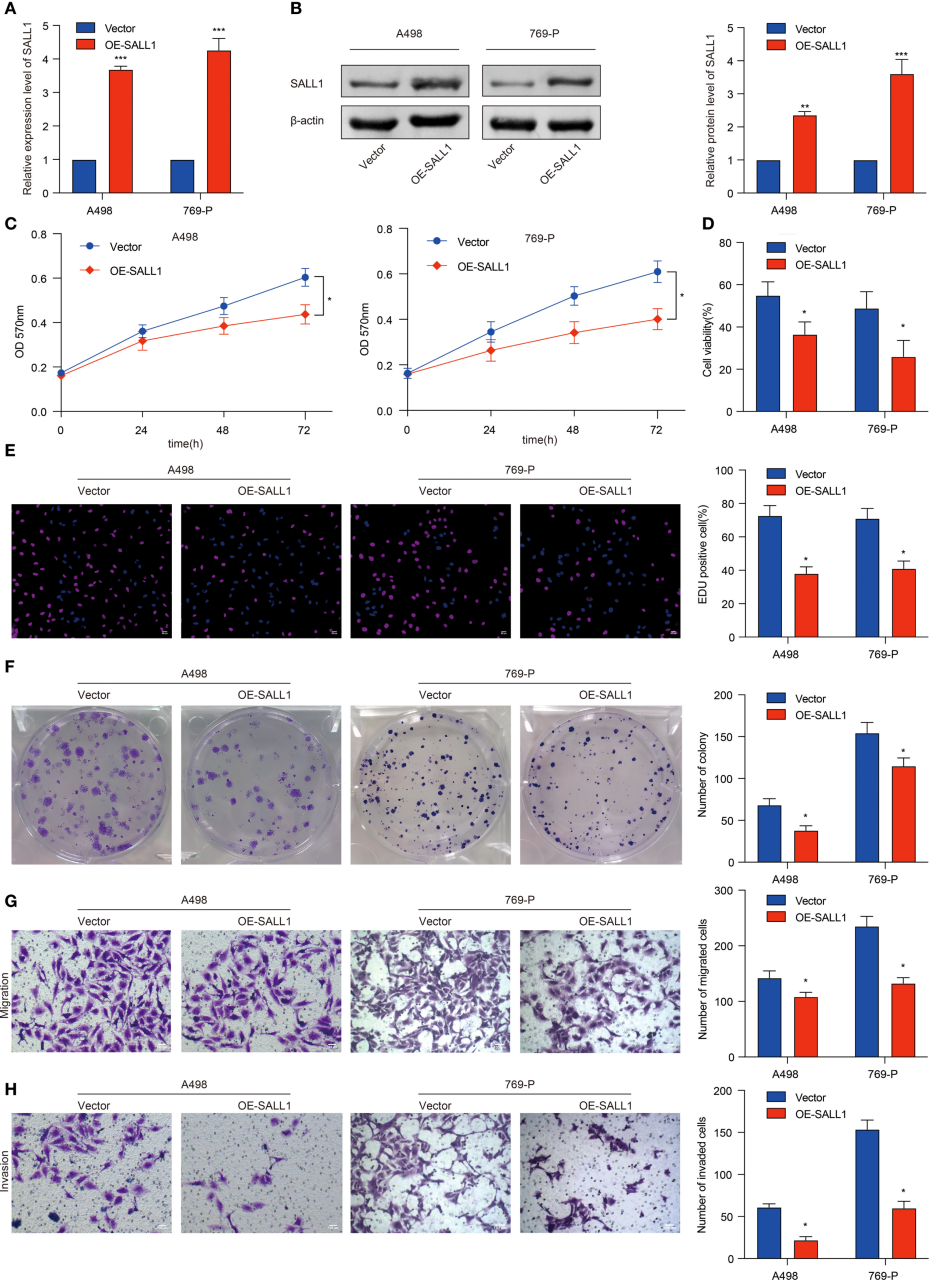
Functional role of SALL1 in ccRCC cell proliferation, migration, and invasion. **(A, B)** Overexpression of SALL1 (OE-SALL1) in A498 and 769-P ccRCC cell lines confirmed by qRT-PCR and Western blot. **(C, D)** MTT and cell viability assays demonstrating reduced proliferation in OE-SALL1 cells. **(E)** EdU assay showing lower proliferation rates in SALL1-overexpressing cells. **(F)** Colony formation assay revealing decreased colony formation in OE-SALL1 cells. **(G, H)** Transwell migration and invasion assays indicating reduced metastatic potential in OE-SALL1 cells (*P < 0.05, **P < 0.01, ***P < 0.001).

To investigate the functional role of SALL1, we overexpressed it (OE-SALL1) in A498 and 769-P ccRCC cell lines. Overexpression efficiency was confirmed via qRT-PCR (**Figure 8A**), showing a significant increase in SALL1 mRNA levels in both OE-SALL1 A498 and 769-P cells compared to vector-transfected controls. Western blot analysis further validated successful overexpression of SALL1 protein (**Figure 8B**), with β-actin as the loading control. MTT assays revealed that overexpressing SALL1 significantly reduced cell proliferation over time (n = 3 per group; P < 0.05) (**Figure 8C**), with a notable decrease in cell viability in OE-SALL1 cells compared to controls (n = 3 per group; P < 0.05) (**Figure 8D**). EdU staining (**Figure 8E**) and colony formation assays (**Figure 8F**) confirmed that SALL1 overexpression led to decreased cell proliferation and fewer colonies. Additionally, Transwell migration and invasion assays showed that OE-SALL1 cells had significantly lower migration and invasion abilities compared to vector control cells (**Figures 8G, H**). Together, these results suggest that SALL1 overexpression inhibits cell proliferation, migration, and invasion in ccRCC cells, supporting its role as a tumor suppressor and a potential therapeutic target in ccRCC.

### Regulation of SALL1 in ccRCC tumor growth

**Figure 9:**
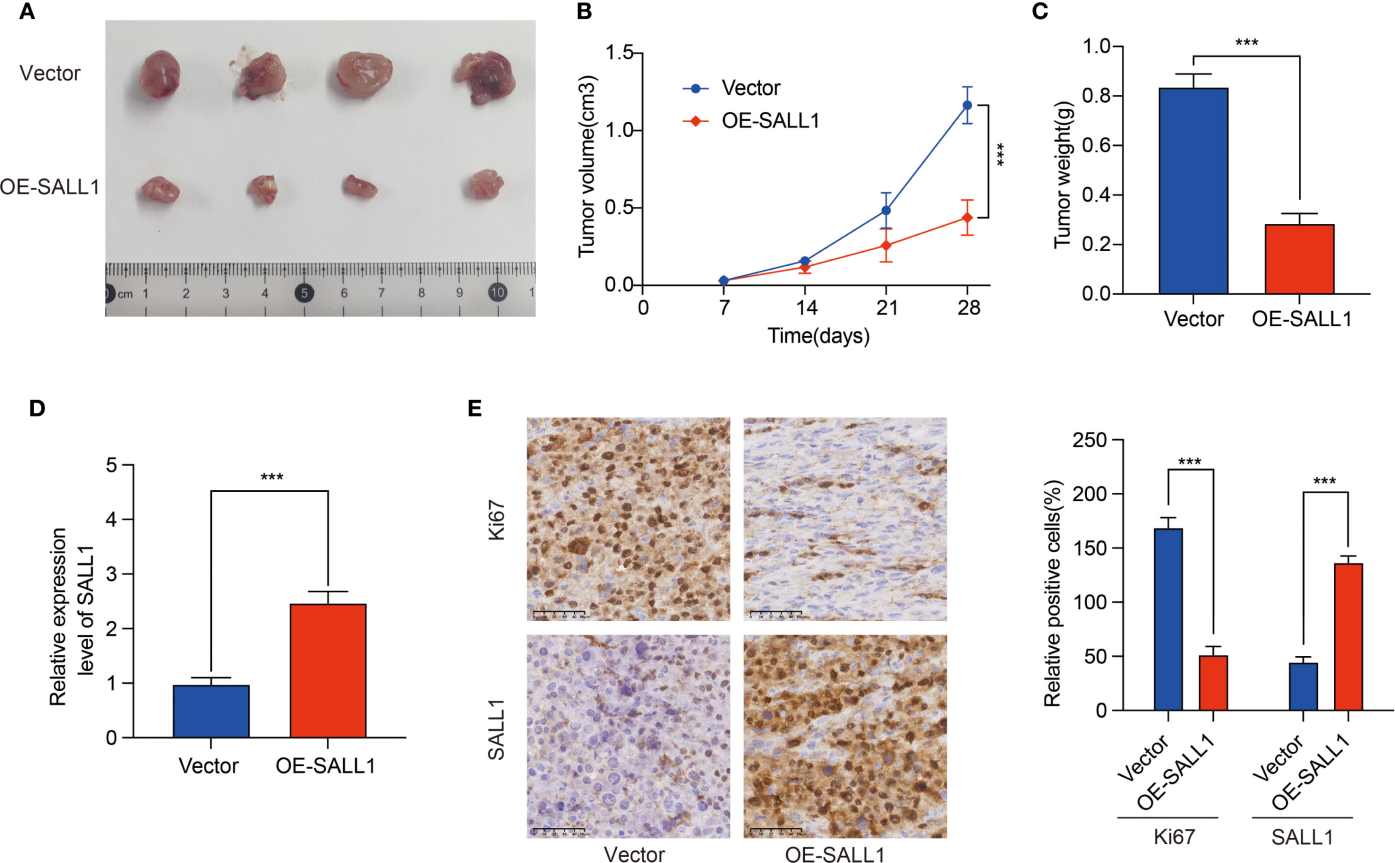
Effect of SALL1 overexpression on tumor growth *in vivo*. **(A)** Tumor size comparison between vector control and OE-SALL1 groups. **(B)** Tumor growth curve. **(C)** Tumor weight. **(D)** qRT-PCR analysis confirming the higher expression of SALL1. **(E)** Immunohistochemistry staining for Ki67 and SALL1. n = 4/group. (***P < 0.001).

To investigate the role of SALL1 in ccRCC tumor growth, we employed a xenograft nude mice model. 769-P cells, transduced with lentiviruses carrying either a vector control or OE-SALL1, were implanted into the nude mice. As shown in **Figure 9**, tumors in the OE-SALL1 group were notably smaller in size (**Figure 9A**), exhibited slower growth (**Figure 9B**), and had reduced weight (**Figure 9C**) compared to the control group. Additionally, SALL1 overexpression was confirmed by qRT-PCR (**Figure 9D**), validating successful transfection. Immunohistochemical staining (**Figure 9E**) showed a significant reduction in Ki67-positive cells, reflecting decreased cell proliferation in the OE-SALL1 group. These results suggest that SALL1 overexpression suppresses ccRCC tumor growth *in vivo*.

## Discussion

In this study, we demonstrated that SALL1 is drastically downregulated in ccRCC tissues and cell lines, and low SALL1 expression is correlated with worse prognosis and later tumor stage. Functional tests revealed overexpression of SALL1 prevents proliferation, migration, and invasion in ccRCC cells, implying a role in acting as a tumor suppressor. Kaplan-Meier survival and Cox multivariate analyses confirmed low SALL1 expression as an independent predictive indicator of worse OS, PFS, and DSS in ccRCC. Importantly, our immune cell infiltration analysis provided complex but informative insights into the immunological roles of SALL1. We found a negative correlation between SALL1 expression and both the ImmuneScore and ESTIMATEScore, suggesting that tumors with lower SALL1 expression exhibit an overall more immunogenic and inflammatory microenvironment. However, when analyzing specific immune cell subsets, high SALL1 expression was positively associated with beneficial anti-tumor immune cells, notably M1 macrophages and resting CD4+ memory T cells, which are indicative of a more effective anti-tumor immune response. Interestingly, our analysis revealed that low SALL1 expression was correlated with a higher overall ImmuneScore, whereas high SALL1 expression was associated with beneficial anti-tumor immune subsets. At first glance, these findings may appear contradictory; however, they likely reflect differences in the qualitative composition of immune infiltration rather than the absolute quantity. A higher ImmuneScore in the low SALL1 group may predominantly represent the enrichment of immunosuppressive or tumor-promoting immune cell populations, such as regulatory T cells, M2 macrophages, or myeloid-derived suppressor cells, which contribute to tumor progression despite elevated overall immune infiltration. In contrast, higher SALL1 expression appears to favor infiltration by functionally beneficial subsets that promote anti-tumor immunity, even if the total immune infiltration is relatively lower. Thus, SALL1 expression may not dictate the overall magnitude of immune infiltration but rather its immunological quality, shifting the balance between pro-tumor and anti-tumor immune populations within the tumor microenvironment. Moreover, drug sensitivity analysis revealed increased SALL1 expression makes cell lines more sensitive to various anticancer agents, implying potential utility in targeted therapy and immune therapy prediction. Overall, these results imply a significant role in ccRCC progression, affecting both tumor behavior and the immune tumor microenvironment, positioning SALL1 as a valuable predictive biomarker and a promising therapeutic target.

Our results are in accordance with existing reports on the role in tumor suppression and immune regulation in other types of malignancies. It has been reported in research that SALL1 is a tumor suppressor in breast cancer and functions in cell senescence and metastasis (14). Genome-scale *in vivo* RNA interference (RNAi) screens revealed a significant role in tumor suppression in human breast cancer and was associated with reduced CDH1 levels, a major player in epithelial-to-mesenchymal transition (EMT) (24). Similarly, in human glioma, SALL1 was reported to inhibit proliferation and metastasis in a role involving the Wnt/β-Catenin signaling pathway (25). Further supporting such a function, LncRNA PART1 was identified to inhibit proliferation and migration in glioma cells via the axis of miR-374b/SALL1. Overexpression of LncRNA PART1 results in overexpression of SALL1 via downregulation and binding with miR-374b, and consequently inhibiting proliferation, migration, and EMT (26). In genitourinary cancers, SALL1 also shows tumor-suppressive roles. In prostate cancer, miR-4286 promotes tumor progression by directly targeting SALL1 (16), while in renal cell carcinoma, LINC00461 regulates SALL1 expression via miR-942, influencing drug resistance and patient survival (27). These findings support our results and indicate that SALL1 dysregulation is part of a broader paradigm across genitourinary tumors. In accordance with these observations, we found in the current study that overexpression of SALL1 significantly inhibited proliferation, migration, and invasion in ccRCC cells, confirming again its tumor-inhibitory function in kidney cancer. Furthermore, epigenetic silencing through promoter hypermethylation of SALL1 was previously demonstrated in a wide array of other malignancies, such as non-small cell lung cancer (28), head and neck squamous cell carcinoma (28), prostate cancer (28), breast cancer (28), colon cancer (28), chronic lymphocytic leukemia (29), and lymphocytic leukemia (30). Although we didn’t investigate epigenetic downregulation of SALL1 in ccRCC, what we found in the current study suggests downregulation could be coupled with such mechanisms and is a matter in need of investigation.

In contrast with current research centered on the role of SALL1 in tumorigenesis, we build on these findings by linking immune cell infiltration and SALL1 expression directly in ccRCC. We observed a direct correlation with immune cell subsets, notably M1 macrophages and CD4+ T cells, suggesting a potential role in modulating the immune microenvironment. This aligns with recent studies on the immune landscape of renal cell carcinoma, where immune cell infiltration plays a crucial role in driving disease progression and treatment outcomes. For instance, research by Chevrier et al (10). demonstrated that immune cell composition, particularly macrophage and T cell infiltration, significantly influences ccRCC progression, which is consistent with our findings. Moreover, our study provides new insights into SALL1’s involvement in regulating immune cell populations, which adds a novel dimension to the understanding of the tumor microenvironment (TME) in ccRCC. While previous studies have focused on immune checkpoint inhibitors and immune cell infiltration (31), our research uncovers the potential of SALL1 to modulate immunity actively. By linking SALL1 expression with specific immune cell subsets, we propose that SALL1 could be a key player in shaping the immune response in ccRCC, potentially impacting immune evasion and the effectiveness of treatments. Additionally, our drug sensitivity analysis revealed that SALL1 expression is positively correlated with increased sensitivity to tyrosine kinase inhibitors (TKIs) and chemotherapeutic agents, a finding not previously reported in the literature. This contrasts with prior studies that primarily focused on immune checkpoint inhibitors or targeted therapies (31–34). Moreover, our findings suggest that SALL1 holds potential for guiding personalized therapy in ccRCC. Given that SALL1 expression was associated with enhanced sensitivity to multiple tyrosine kinase inhibitors and chemotherapeutic agents, patients with high SALL1 expression may benefit more from these targeted regimens. Conversely, low SALL1 expression, which correlated with an immunosuppressive tumor microenvironment, might indicate a patient subgroup more likely to respond to immune checkpoint blockade or combination therapies. Thus, SALL1 could serve not only as a prognostic biomarker but also as a stratification marker to personalize treatment strategies, optimizing therapeutic efficacy while minimizing unnecessary toxicity. Future prospective clinical studies are warranted to validate the role of SALL1 in therapy selection and to explore its integration into biomarker-driven clinical decision-making in ccRCC.

One of the strengths of our study is the comprehensive analysis of immune cell infiltration in ccRCC. Using advanced immune algorithms such as ESTIMATE, ImmuneScore, and StromalScore, we assessed the immune landscape in relation to SALL1 expression, a topic that has been underexplored in previous research. While most studies on SALL1 have focused on its role in tumor biology (10, 35–37), we emphasize its modulatory impact on the TME. Consistent with recent findings, our results show that higher SALL1 expression correlates with increased infiltration of M1 macrophages and CD4+ T cells, suggesting that SALL1 may help create a more favorable immune environment, enhancing anti-tumor immune responses. Furthermore, we used a novel drug sensitivity analysis with the pRRophetic package, which is especially useful in personalized treatment strategies for ccRCC. Although drug sensitivity profiling has been applied in other cancers, its use in ccRCC, particularly in relation to SALL1 expression, has not been well explored. Our results indicate that higher SALL1 expression is associated with increased sensitivity to various anticancer agents, including TKIs and chemotherapy drugs, suggesting that SALL1 may be a valuable predictive biomarker for therapeutic response. Additionally, recent studies in prostate cancer have highlighted SALL1’s potential in predicting therapeutic responses (38), reinforcing its potential not only in ccRCC but also across other cancer types. These findings further underscore the importance of SALL1 in modulating immune responses and drug sensitivity, laying a strong foundation for its future clinical application in optimizing treatment regimens for ccRCC patients.

Although this study provides valuable insights into the role of SALL1 in ccRCC, several limitations should be addressed in future research. Firstly, while our findings are based on publicly available data and patient samples, further prospective clinical studies are necessary to validate the prognostic value of SALL1 in ccRCC. Secondly, the sample size of patient specimens in our study was relatively small, which may limit the generalizability of the results. Future studies with larger and multi-center patient cohorts are needed to strengthen the robustness and clinical applicability of our findings. Thirdly, although we demonstrated significant associations between SALL1 expression, immune infiltration, and drug sensitivity, the underlying molecular mechanisms remain insufficiently explored. Future studies employing transcriptomic, epigenetic, and CRISPR-based functional analyses are warranted to delineate how SALL1 modulates the immune microenvironment and drug responsiveness. Fourthly, our study primarily relied on bioinformatics tools and *in vitro* assays, so the *in vivo* therapeutic effects of SALL1 overexpression should be validated in larger animal models and clinical trials. Additionally, we recognize a significant limitation related to our *in vivo* xenograft studies. These experiments were conducted using immunocompromised nude mice, which inherently lack a functional adaptive immune system. Consequently, this model precludes direct evaluation of SALL1’s immunomodulatory effects within the tumor microenvironment *in vivo*. Future investigations using immunocompetent syngeneic models or humanized mouse systems will be essential to comprehensively assess how SALL1 regulates immune cell dynamics and anti-tumor immunity. Moreover, the associations between SALL1 expression and immune infiltration or drug sensitivity observed in our bioinformatic analyses remain correlative rather than causal. Functional validation in immune-competent preclinical models and prospective patient cohorts is required to substantiate SALL1’s predictive value for immunotherapy response or targeted therapy efficacy. Lastly, while our analysis suggests that SALL1 may modulate immune responses, the specific immune subsets involved and the potential for SALL1-based immunotherapeutic strategies warrant further investigation. These limitations highlight the need for future studies to integrate mechanistic experiments, diverse animal models, and clinical validation to fully explore the therapeutic potential of SALL1 in ccRCC.

## Conclusions

In conclusion, this study underscores the pivotal role of SALL1 in ccRCC and highlights its potential as both a prognostic biomarker and a therapeutic target. The observed downregulation of SALL1 in ccRCC, coupled with its association with tumor progression and immune modulation, suggests its crucial role in shaping the tumor microenvironment. Furthermore, given its correlation with immune cell infiltration and drug sensitivity, SALL1 holds promise as a valuable marker for developing personalized therapeutic strategies in ccRCC.

## Data Availability

The RNA-seq data and clinical information were obtained from The Cancer Genome Atlas (TCGA, https://portal.gdc.cancer.gov/) database. The datasets analyzed for this study are deposited in the Gene Expression Omnibus (GEO, https://www.ncbi.nlm.nih.gov/geo/), accession numbers GSE53757 and GSE66272. The Western blot and qRT-PCR experimental data generated by the authors from ccRCC tissues are available upon reasonable request. Additionally, all other relevant data, including the results of the bioinformatics analyses, can be made available upon reasonable request to the corresponding authors.

## References

[1] Huang Q Yang J Liu GX Zi H Tang SD Jia HC. Changes in disease burden and global inequalities in bladder, kidney and prostate cancers from 1990 to 2019: a comparative analysis based on the global burden of disease study 2019. BMC Public Health. (2024) 24:891. doi: 10.1186/s12889-024-18353-9, PMID: 38528465 PMC10962085

[2] Robila V Kraft AO Smith SC. New entities, new technologies, new findings: A review of the cytologic features of recently established subtypes of renal cell carcinoma. Cancer Cytopathol. (2019) 127:79–97. doi: 10.1002/cncy.22093, PMID: 30690877

[3] Prasad SR Humphrey PA Catena JR Narra VR Srigley JR Cortez AD. Common and uncommon histologic subtypes of renal cell carcinoma: imaging spectrum with pathologic correlation. Radiographics. (2006) 26:1795–806. doi: 10.1148/rg.266065010, PMID: 17102051

[4] Kuthi L Jenei A Hajdu A Németh I Varga Z Bajory Z. Prognostic factors for renal cell carcinoma subtypes diagnosed according to the 2016 WHO renal tumor classification: a study involving 928 patients. Pathol Oncol Res. (2017) 23:689–98. doi: 10.1007/s12253-016-0179-x, PMID: 28032311

[5] Bedke J Albiges L Capitanio U Giles RH Hora M Ljungberg B. The 2022 updated european association of urology guidelines on the use of adjuvant immune checkpoint inhibitor therapy for renal cell carcinoma. Eur Urol. (2023) 83:10–4. doi: 10.1016/j.eururo.2022.10.010, PMID: 36511268

[6] Shi X Feng D Li D Zhang F Wei W. The role of lymph node dissection for non-metastatic renal cell carcinoma: an updated systematic review and meta-analysis. Front Oncol. (2021) 11:790381. doi: 10.3389/fonc.2021.790381, PMID: 35096589 PMC8790094

[7] Bahadoram S Davoodi M Hassanzadeh S Bahadoram M Barahman M Mafakher L. Renal cell carcinoma: an overview of the epidemiology, diagnosis, and treatment. G Ital Nefrol. (2022) 39:2022–33., PMID: 35819037

[8] Gebrael G Sahu KK Agarwal N Maughan BL. Update on combined immunotherapy for the treatment of advanced renal cell carcinoma. Hum Vaccin Immunother. (2023) 19:2193528. doi: 10.1080/21645515.2023.2193528, PMID: 37062953 PMC10114990

[9] Barata PC Rini BI. Treatment of renal cell carcinoma: Current status and future directions. CA Cancer J Clin. (2017) 67:507–24. doi: 10.3322/caac.21411, PMID: 28961310

[10] Chevrier S Levine JH Zanotelli VRT Silina K Schulz D Bacac M. An immune atlas of clear cell renal cell carcinoma. Cell. (2017) 169:736–749.e18. doi: 10.1016/j.cell.2017.04.016, PMID: 28475899 PMC5422211

[11] Hosoe-Nagai Y Hidaka T Sonoda A Sasaki Y Yamamoto-Nonaka K Seki T. Re-expression of Sall1 in podocytes protects against adriamycin-induced nephrosis. Lab Invest. (2017) 97:1306–20. doi: 10.1038/labinvest.2017.69, PMID: 28759006

[12] Exner CRT Kim AY Mardjuki SM Harland RM. sall1 and sall4 repress pou5f3 family expression to allow neural patterning, differentiation, and morphogenesis in Xenopus laevis. Dev Biol. (2017) 425:33–43. doi: 10.1016/j.ydbio.2017.03.015, PMID: 28322736

[13] Misawa K Misawa Y Imai A Mochizuki D Endo S Mima M. Epigenetic modification of SALL1 as a novel biomarker for the prognosis of early stage head and neck cancer. J Cancer. (2018) 9:941–9. doi: 10.7150/jca.23527, PMID: 29581773 PMC5868161

[14] Ma C Wang F Han B Zhong X Si F Ye J. SALL1 functions as a tumor suppressor in breast cancer by regulating cancer cell senescence and metastasis through the NuRD complex. Mol Cancer. (2018) 17:78. doi: 10.1186/s12943-018-0824-y, PMID: 29625565 PMC5889587

[15] Yuan J Li G Zhong F Liao J Zeng Z Ouyang S. SALL1 promotes proliferation and metastasis and activates phosphorylation of p65 and JUN in colorectal cancer cells. Pathol Res Pract. (2023) 250:154827. doi: 10.1016/j.prp.2023.154827, PMID: 37741137

[16] Li Z Zhao S Wang H Zhang B Zhang P. miR-4286 promotes prostate cancer progression by targeting the expression of SALL1. J Gene Med. (2023) 25:e3127. doi: 10.1002/jgm.3127, PMID: 31693770

[17] Wang Y Li Y Li X. Ultrasound microbubble-mediated miR-503-5p downregulation suppressed *in vitro* CRC progression via promoting SALL1 expression. Tissue Cell. (2022) 76:101811. doi: 10.1016/j.tice.2022.101811, PMID: 35567907

[18] Zhang G Song C Yin M Liu L Zhang Y Li Y. TRAPT: a multi-stage fused deep learning framework for predicting transcriptional regulators based on large-scale epigenomic data. Nat Commun. (2025) 16:3611. doi: 10.1038/s41467-025-58921-0, PMID: 40240358 PMC12003887

[19] Li Z Fan J Xiao Y Wang W Zhen C Pan J. Essential role of Dhx16-mediated ribosome assembly in maintenance of hematopoietic stem cells. Leukemia. (2024) 38:2699–708. doi: 10.1038/s41375-024-02423-3, PMID: 39333759

[20] Hu M Yuan X Liu Y Tang S Miao J Zhou Q. IL-1β-induced NF-κB activation down-regulates miR-506 expression to promotes osteosarcoma cell growth through JAG1. BioMed Pharmacother. (2017) 95:1147–55. doi: 10.1016/j.biopha.2017.08.120, PMID: 28926924

[21] Liang L Liang X Yu X Xiang W. Bioinformatic Analyses and Integrated Machine Learning to Predict prognosis and therapeutic response Based on E3 Ligase-Related Genes in colon cancer. J Cancer. (2024) 15:5376–95. doi: 10.7150/jca.98723, PMID: 39247594 PMC11375543

[22] Ritchie ME Phipson B Wu D Hu Y Law CW Shi W. limma powers differential expression analyses for RNA-sequencing and microarray studies. Nucleic Acids Res. (2015) 43:e47. doi: 10.1093/nar/gkv007, PMID: 25605792 PMC4402510

[23] Yu G Wang LG Han Y He QY. clusterProfiler: an R package for comparing biological themes among gene clusters. Omics. (2012) 16:284–7. doi: 10.1089/omi.2011.0118, PMID: 22455463 PMC3339379

[24] Wolf J Müller-Decker K Flechtenmacher C Zhang F Shahmoradgoli M Mills GB. An *in vivo* RNAi screen identifies SALL1 as a tumor suppressor in human breast cancer with a role in CDH1 regulation. Oncogene. (2014) 33:4273–8. doi: 10.1038/onc.2013.515, PMID: 24292671 PMC6662585

[25] Chi D Zhang W Jia Y Cong D Hu S. Spalt-like transcription factor 1 (SALL1) gene expression inhibits cell proliferation and cell migration of human glioma cells through the wnt/β-catenin signaling pathway. Med Sci Monit Basic Res. (2019) 25:128–38. doi: 10.12659/MSMBR.915067, PMID: 31040265 PMC6511114

[26] Deng YW Shu YG Sun SL. LncRNA PART1 inhibits glioma proliferation and migration via miR-374b/SALL1 axis. Neurochem Int. (2022) 157:105347. doi: 10.1016/j.neuint.2022.105347, PMID: 35490895

[27] Chen Y He J Su C Wang H Chen Y Guo W. LINC00461 affects the survival of patients with renal cell carcinoma by acting as a competing endogenous RNA for microRNA−942. Oncol Rep. (2019) 42:1924–34. doi: 10.3892/or.2019.7311, PMID: 31545458 PMC6775798

[28] Hill VK Hesson LB Dansranjavin T Dallol A Bieche I Vacher S. Identification of 5 novel genes methylated in breast and other epithelial cancers. Mol Cancer. (2010) 9:51. doi: 10.1186/1476-4598-9-51, PMID: 20205715 PMC2841122

[29] Zhang C Zhao H Li J Liu H Wang F Wei Y. The identification of specific methylation patterns across different cancers. PloS One. (2015) 10:e0120361. doi: 10.1371/journal.pone.0120361, PMID: 25774687 PMC4361543

[30] Kalari S Pfeifer GP. Identification of driver and passenger DNA methylation in cancer by epigenomic analysis. Adv Genet. (2010) 70:277–308. doi: 10.1016/B978-0-12-380866-0.60010-1, PMID: 20920752 PMC2951285

[31] Topalian SL Hodi FS Brahmer JR Gettinger SN Smith DC McDermott DF. Safety, activity, and immune correlates of anti-PD-1 antibody in cancer. N Engl J Med. (2012) 366:2443–54. doi: 10.1056/NEJMoa1200690, PMID: 22658127 PMC3544539

[32] Liu J Xu J Zhang T Xu K Bao P Zhang Z. Decoding the immune microenvironment of clear cell renal cell carcinoma by single-cell profiling to aid immunotherapy. Front Immunol. (2022) 13:791158. doi: 10.3389/fimmu.2022.791158, PMID: 35812372 PMC9263726

[33] Lu C Wang Y Nie L Chen L Li M Qing H. Comprehensive analysis of cellular senescence-related genes in the prognosis, tumor microenvironment, and immunotherapy/chemotherapy of clear cell renal cell carcinoma. Front Immunol. (2022) 13:934243. doi: 10.3389/fimmu.2022.934243, PMID: 36189255 PMC9523431

[34] Wang Y Wang Y Liu B Gao X Li Y Li F. Mapping the tumor microenvironment in clear cell renal carcinoma by single-cell transcriptome analysis. Front Genet. (2023) 14:1207233. doi: 10.3389/fgene.2023.1207233, PMID: 37533434 PMC10392130

[35] Linehan WM Ricketts CJ. The Cancer Genome Atlas of renal cell carcinoma: findings and clinical implications. Nat Rev Urol. (2019) 16:539–52. doi: 10.1038/s41585-019-0211-5, PMID: 31278395

[36] Wang K Ding Y Liu Y Ma M Wang J Kou Z. CPA4 as a biomarker promotes the proliferation, migration and metastasis of clear cell renal cell carcinoma cells. J Cell Mol Med. (2024) 28:e18165. doi: 10.1111/jcmm.18165, PMID: 38494845 PMC10945090

[37] Xu Z Wu Y Fu G Chen X Sun J Tian J. SAA1 has potential as a prognostic biomarker correlated with cell proliferation, migration, and an indicator for immune infiltration of tumor microenvironment in clear cell renal cell carcinoma. Int J Mol Sci. (2023) 24:7505. doi: 10.3390/ijms24087505, PMID: 37108666 PMC10138873

[38] Wang Y Zhang X Wu L Feng Q Luo Z Zeng T. A necroptosis gene signature predicts prostate cancer recurrence, and is linked to somatic mutation, therapeutic landscape, and immune infiltration. Am J Transl Res. (2023) 15:2460–80., PMID: 37193176 PMC10182515

